# Piano level laser therapy versus epidermal growth factor injection for painful myogenic temporomandibular disorder (a randomized clinical trial)

**DOI:** 10.1007/s00784-025-06189-5

**Published:** 2025-02-06

**Authors:** Passant Osama Qataya, Azza Mohamed Zaki, Fatma Amin, Ahmed Swedan, Hagar Elkafrawy

**Affiliations:** 1 Oral Medicine, Periodontology, Radiology and Diagnosis, Department of Oral Medicine, Periodontology, Radiology and Diagnosis, Faculty of Dentistry, Alexandria University, Alexandria, Egypt; 2 Oral and Maxillofacial Surgery, Department of Oral and Maxillofacial Surgery, Faculty of Dentistry, Alexandria University, Alexandria, Egypt; 3 Medical Biochemistry, Department of Medical Biochemistry, Faculty of Medicine, Alexandria University, Alexandria, Egypt

**Keywords:** Photobiomodulation, HILT, Injection, Non-invasive

## Abstract

**Objective:**

The aim of this clinical trial was to evaluate the effectiveness of Piano level laser therapy using Nd-YAG laser and intramuscular EGF injection in pain alleviation, function, and quality of life improvement in patients suffering from myogenic TMD.

**Materials and methods:**

A randomized clinical trial was performed on 29 patients suffering from chronic painful myogenic TMD based on diagnostic criteria for temporomandibular disorders. Group I (*n* = 13patients) was treated using 1064 nm Nd-YAG Laser (4 sessions once/week). Group II (*n* = 14 patients) was treated by intramuscular injection of EGF. Pain using numerical rating score, pain free opening and unassisted maximum opening were measured at baseline, 7,14,21 days, 1 and 3 months. Quality of life using OHIP-14 was assessed at baseline, 1 and 3 months.

**Results:**

Results showed that there was a significant pain reduction (*P* **< 0.000**) and increase in pain free opening (P **< 0.0001**) in both test groups. However, only group I showed a significant increase in maximum opening (*P* = **0.007**). Quality of life significantly improved in both groups (*P* = **0.0001**). There was no significant difference between the two treatments in pain scores, pain free opening, maximum opening nor quality of life.

**Conclusion:**

Both treatment modalities offered effective and cost-effective non- to minimally invasive treatment options for myogenic TMD with no side effects.

**Clinical relevance:**

Myogenic TMD forms a public health issue and is a common musculoskeletal problem causing pain and disability. The proposal of effective, non-invasive, and affordable treatment options can help solve this issue.

**Supplementary Information:**

The online version contains supplementary material available at 10.1007/s00784-025-06189-5.

## Introduction

Temporomandibular disorder (TMD) is an umbrella term used to describe a heterogenous group of painful and non-painful disorders affecting the temporomandibular joint (TMJ), associated muscles of mastication and related structures [1]. According to Diagnostic Criteria for TMD (DC/TMD) Axis I, TMDs are divided into muscle disorders (including myofascial pain with and without mouth-opening limitation); disc displacement with or without reduction and mouth-opening limitation, arthralgia, arthritis, and arthrosis [2]. Temporomandibular disorders (TMDs) form a public health issue affecting around 31% of adults and 11% of adolescents and children in the general population [3]. They are considered the second most common musculoskeletal problem (after chronic lower back pain) causing pain and disability [2] and the most common cause of chronic orofacial pain [3] which have a significant impact on the quality of life, psychological functions and individual’s daily activity [2, 4]. The most common condition among TMDs is myogenic TMD (M TMD) affecting 45.3% of TMD cases [3]. Patients suffering from M TMD usually complain of dull aching pain in the masticatory apparatus, that can be unilateral or bilateral. The pain is typically felt in and around the ear, angle/body of the mandible and the temple area. There can also be limitation in mouth opening and deviation of the mandible while opening [4]. The most affected muscles are the masseter followed by the temporalis [5], both of which are the basis during clinical diagnosis [2].

The pathophysiology of M TMD is unclear [4, 5]. It is suggested to involve a myriad of interactions between genetic, biological and psychosocial parameters; thus, it is termed a “complex biopsychosocial disorder” [4]. Central and peripheral sensitization play an evident role in M TMD development [6]. Repetitive muscle straining due to parafunctional habits, stress or anxiety can lead to muscle injury due to induction of ischemia, hypoxia, decreased pH, and accumulation of inflammatory mediators and algogenic substances such as serotonin, bradykinin, calcitonin gene-related peptide (CGRP), nerve growth factor (NGF), histamine, substance P and glutamate. The accumulation of inflammatory and pain mediators leads to a state of oxidative stress and sensitization of muscle nociceptors respectively [6, 7]. This leads to persistent muscle fiber shortening and trigger point activation (energy crisis- muscle contraction cycle) [8].

Due to this complex pathophysiology of M TMD, many treatment modalities have been proposed with the aim of eliminating pain, improving function and quality of life. Non-surgical treatment modalities are considered the first line of treatment for TMDs, whether of myogenic or articular origin [9]. These treatment modalities include acupuncture, occlusal splints, therapeutic jaw exercises, physiotherapy, postural training, pharmacotherapy, transcutaneous electrical nerve stimulation (TENS), and behavioral therapies [10].

Laser photo-biomodulation (optical window 600–1200 nm) has gained popularity in pain therapy and treatment of chronic painful musculoskeletal disorders, including TMDs [10–12]. This is due to its anti-inflammatory, immunomodulatory, bioregenerative and analgesic actions, in addition to its low cost and being non-invasive [10–12]. The exact mechanism of action of photo-biomodulation is still not fully understood. However, it is thought that it can affect the synthesis and release of several substances involved in analgesia, such as increasing beta-endorphins, which can inhibit pain sensations, decreasing substance P, which causes hyperalgesia, and reducing the release of histamine and bradykinin in injured tissues leading to increased pain threshold [11, 12]. It also causes an increase in lymphatic flow and blood circulation, reduction of oxidative stress and lipid peroxidation products causing muscle relaxation [10, 11].

Low-level laser therapy (LLLT) and high-intensity laser therapy (HILT) have both been used in photo-biomodulation, pain therapy and in decreasing functional disabilities in chronic musculoskeletal disorders [12]. However, HILT has more powerful beams (> 500 MW) that can penetrate deeper and reach a wider area delivering the desired energy to deeply seated tissues, muscles, tendons and joints [12, 13]. In addition, higher powers can directly act on nerves inhibiting pain signals [14]. Piano level laser therapy (PLLT), a patency delivered by Fotona^®^, enables delivering different levels of high intensity laser using large spot sizes in a stamping technique. It was reported that applying this combined cold and warm PLLT on a patient suffering from pain and paresthesia in the right shoulder has given positive results regarding pain alleviation and range of shoulder motion [14].

Needling therapies, whether dry or wet, are other appealing modes of treatment for myogenic TMDs. Wet needling techniques refer to local injection of substances such as saline, local anesthesia, botulinum toxin, growth factors, corticosteroids or sclerosants intramuscular. Whereas, dry needling is limited to the intramuscular insertion of a monofilament needle to inactivate tender or trigger points [15]. Plasma-rich growth factors (PRGF) [16] have been tested in alleviating the signs and symptoms associated with myogenic TMD. Epidermal growth factor (EGF) is considered one of the constituents of PRGF along with other growth factors such as: platelet-derived growth factor (PDGF), transforming growth factor (TGF-β), vascular endothelial growth factor (VEGF), and nerve growth factor (NGF). All of the aforementioned growth factors play a role in neo-angiogenesis, muscle regeneration and reducing chronic pain [16].

Epidermal growth factor is a mitogenic polypeptide found in platelets and is considered a key regulator in tissue proliferation, wound repair, and enhancing sarcomere structure formation in myogenic cell cultures [17]. EGF has shown to repair muscle damage, increase muscle quality through direct promotion of muscle cells proliferation and differentiation, and regulating the inflammatory response [18]. A clinical trial on diabetic patients suffering from chronic foot ulcers showed that EGF intra-lesional injection promotes healing in those ulcers and prevents foot amputation [19]. It has also increased the proliferation of keratinocytes and fibroblasts damaged by radiation, promoting healing in patients suffering from radiation-induced mucositis [20].

This study aims to evaluate and compare the effect of Piano Level Laser Therapy (PLLT) versus EGF injection as non-to-minimally invasive treatment modalities for alleviating signs and symptoms of M TMD. Additionally, it assesses the impact of these treatments on the quality of life in patients suffering from M TMD.

## Materials and methods

### Study design

A randomized, single-blinded single center clinical trial with 2 parallel groups was carried out. The study fulfilled the CONSORT guidelines [21]. The PICOT question was: Will patients suffering from chronic painful myogenic TMD (Population; P) when treated by PLLT (Intervention; I) in comparison with intramuscular injection of EGF (Comparison; C) show reduction in pain scores (Outcome; O) when followed up at 7, 14, 21 days and at 4 and 12 weeks (Time; T)?

### Study setting

The study was conducted at Oral medicine, Periodontology, Diagnosis and Radiology Department, Faculty of Dentistry, Alexandria University, Egypt. Participants that were enrolled to group I (PLLT) were treated at Laser unit, faculty of Dentistry, Alexandria University.

### Participants

This study was conducted on twenty-nine patients suffering from chronic M TMD pain who were refractory to other conservative treatments.

### Study sample and sample size estimation

The sample size was estimated assuming a 5% alpha error and 80% study power. The mean percent decrease in pain after 6 months was 21.625% for the growth factor group [22] and it was estimated to be 25.53% for the high-intensity laser group [23]. Based on the difference between independent means using the highest SD = 3.3 [23] to ensure enough power, the minimum sample size was calculated to be 13 patients per group, increased to 15 patients per group to compensate for lost to follow up cases. Total sample = number per group x number of groups = 15 × 2 = 30 patients.

### Software

The sample size was based on Rosner’s method [24] calculated by G*Power 3.1.9.7 [25].

### Ethical considerations

This clinical trial received the approval of the ethical committee of Faculty of Dentistry, Alexandria University in December 2022. This clinical trial was registered at ClinicalTrials.gov Identifier: NCT06044974. An informed written consent was obtained from each patient before the start of the clinical procedures. Participants of this clinical trial were treated according to the principles of the modified Helsinki’s code for human clinical studies [26].

### Eligibility criteria

#### Inclusion criteria

1. Patients, both males and females, 20 years and older were included in this clinical trial.
2. Patients suffering from myogenic TMD (myogenic TMD/ Myofascial pain without referral/ Myofascial pain with referral) based on Diagnostic criteria for diagnosis of Temporomandibular joint disorders (DC/TMD) criteria [2].
3. Patients suffering from unilateral or bilateral chronic pain (> 3 months duration) related to masseter and/or temporalis muscles [27].
4. Patients who have not responded to conservative modes of treatment (analgesics, muscle relaxants, fomentation, splints, physiotherapy).

#### Exclusion criteria

1. Patients suffering from any condition that could alter pain sensitivity; neurological diseases, pain of dental origin, pregnancy or lactation, high blood pressure, diabetes mellitus, rheumatic inflammatory disease, fibromyalgia, obstructive sleep apnea, skin infection over injection areas related to masseter and temporalis muscles, and restrictions for the use of laser (pacemakers) [7, 28, 29].
2. Participants who were on medications that can affect pain sensitivity and perception; anticoagulants, analgesics, and antidepressants during the last 2 weeks before the study [7, 29].

### Randomization and allocation concealment

Participants were randomly allocated to one of two groups using a computer-generated random list [30]. The participant allocation lists were kept in opaque, sealed envelopes and arranged sequentially by a dental assistant who was not involved in the study. Each envelope was opened at the time of intervention [31].

### Blinding

Data assessors, including an oral medicine co-worker, and a statistician were blinded to the treatment patient each patient had received. Patients and primary clinician were not blinded to patients’ treatment. This is a single-blinded clinical trial.

### Intervention

#### Group I (*n* = 13)

fourteen patients suffering from myogenic TMD were allocated into this group. One patient dropped out and did not finish the PLLT sessions nor come for follow-up.

Patients in this group received four combined PLLL sessions using Fotona LightWalker^®^1064 nm Nd-YAG laser (once/week) [32, 33] utilizing Geneova handpiece (focal spot size = 11 mm) that is characterized by its unique design with a collimated flat-top radiance profile thus the uniform delivery of high peak power. Each treatment session included two passes using Geneva handpiece in a non-contact mode. The first pass was performed using “cold” Piano Level Laser Therapy “PLLT” settings for photo biomodulation – MSP pulse (100 µs), *P* = 2 W (Ps = 0.21 W/cm^2^), 10 Hz, with 1 min treatment duration per spot. A second pass, higher “warm” PLLT settings that caused mild heating of the tissue, aimed at pain relief – MSP pulses 5 W (Ps = 0.55 W/cm^2^), 60 Hz, where the handpiece was held at each spot from 1 to 2 minutes, depending on patient heat tolerance [14]. Both the masseter and temporalis muscles were divided into three spots: insertion, body, and origin for the masseter muscle and anterior, middle, and posterior for temporalis muscle. A stamping technique was carried out for both passes. However, when treating temporal area beyond hairline specially in female patients, the duration of treatment was reduced to 30 s to 1 min specially during the second pass. A little movement back and forth in the same zone, i.e. anterior, middle, or posterior was performed to reduce heat sensation due to the presence of hair.

#### Group II (*n* = 14)

fifteen patients suffering from myogenic TMD were enrolled in group II. One patient dropped out after the 1st injection and did not come to follow-up.

Epidermal Growth factor (EGF) manufactured by DERMAQUEL PARIS^®^ injection into masseter and temporalis muscles of patients allocated to this group. A 1.0 ml, ½ inch (12.7 mm), 31-gauge insulin syringe was used for intramuscular injection. Each muscle was divided into 3 zones; origin, body and insertion for masseter muscle, anterior, middle, and posterior for temporalis muscle [2]. For each muscle, a 3-point injection technique was performed: 1 injection point in each zone corresponding to painful trigger point [34]. Each point received 0.1 ml of EGF [35]. The patients enrolled in this group were injected twice at baseline and day 14.

### Outcome measures

(A) **Clinical outcomes**
  Subjective Pain score as measured by Numerical Rating Scale (NRS) [36]. Pain was measured at baseline, 7,14,21 days, and 1- and 3-months.
  Maximum unassisted opening (MO) and pain free opening (PFO) [2] at baseline, 7, 14, 21 days, and 1- and 3-months.
(B) **Quality of Life using the Oral Health Impact Profile (OHIP)-14 questionnaire** [37] at baseline, 4 and 12 weeks.

### Statistical analysis

The clinical variables (NRS, PFO, MO) and OHIP-14 scores were checked for normality using Kolmogorov-Smirnov, Shapiro-Wilk, and Histogram curves. Data that deviated from normality were square root and log transformed. Because the data did not satisfy the normality assumption even after transformation, Friedman’s analysis was used to test significant variations in the repeatedly measured different parameters over time (intragroup analyses). The null hypothesis was that the distribution of the variables is the same across different time categories. Following Friedman’s analyses, the post-hoc Wilcoxon test was used to compare variables, rejecting their null hypothesis. The Wilcoxon test fits the non-parametric data and has a higher power to detect change than Dunn-Bonferroni [38, 39].

The non-parametric Mann-Whitney test was selected to examine significant differences between the two test groups (intergroup); PLLT and EGF injection, based on the null hypothesis that the distribution of each variable was the same between treatment categories. Significance was reported at level α ≤ 0.05. SPSS version 21 software was used for these analyses. To test for significant associations among the non-normally distributed data (NRS, PFO, MO, OHIP), Spearman’s rank correlation analysis (Spearman’s rho) was employed, with a significance level set at α ≤ 0.05. The analysis was performed on data collected before treatment (day zero), as well as at the 1-month and 3-month follow-ups.

## Results

**Fig. 1:**
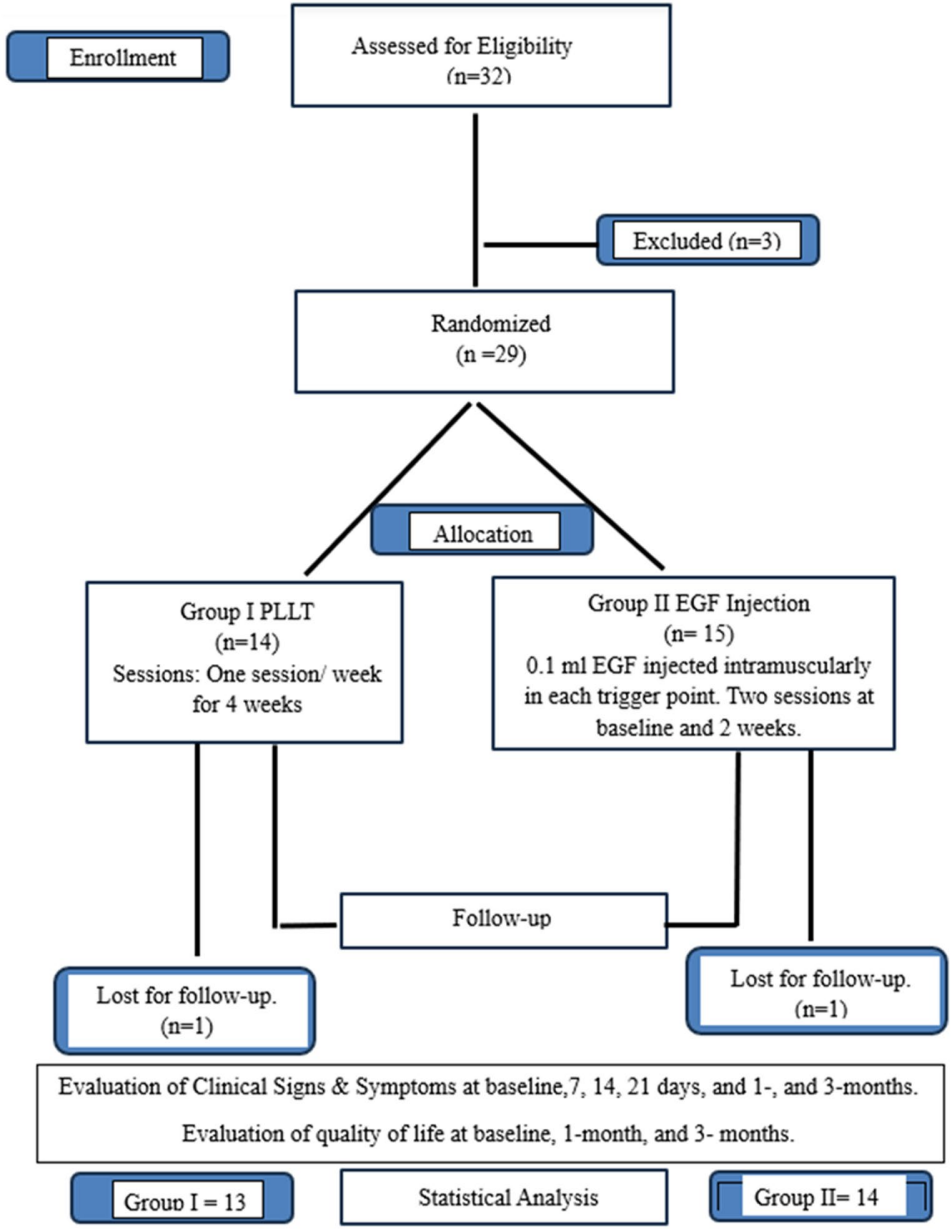
Flow diagram following consort’s guidelines (2010)

Thirty-two patients (28 females and 4 male patients) were assessed for eligibility criteria between December 2022 and October 2023. Only 29 patients (25 females and 4 male patients) participated in this randomized clinical trial. Two patients dropped out: one during receiving PLLT sessions and one patient after receiving the 1st EGF intramuscular injection. A total of 27 patients completed this clinical trial, as shown in Fig. 1, following the consort’s 2010 flow diagram recommendations [40]. The mean age of all participants of this clinical trial is (27.74); for group I (28.23 ± 7.07) and for group II (27.30 ± 7.25) (Table 1). Results of Mann-Whitney test show that there is no significant difference between the age of participants in the two test groups.

**Table 1:** Descriptive demographic data of participants of the clinical trial regarding age and gender of each group

Gender	*n*	Group I (Laser)	Group II (EGF)
Male	4	1	3
Female	25	13	12
Age	27. 74 ± 6.95	28.23 ± 7.07	27.30 ± 7.25

Group I received Piano Level Laser Therapy (PLLT), and group II received epidermal growth factor (EGF) injection. n = number of patients in each treatment group. Data are presented as mean ± SD

### Effects of treatment modalities on clinical pain scores using Numerical Rating Scale (NRS)

**Fig. 2:**
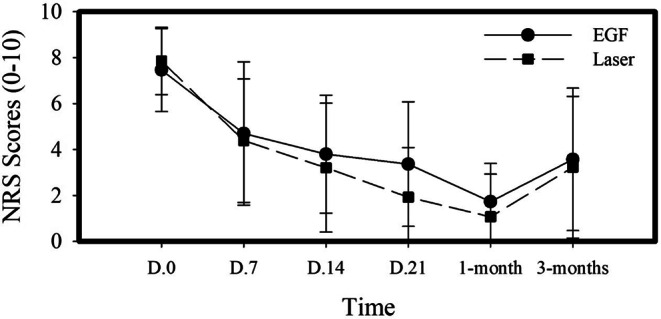
Effect of treatment modalities on clinical pain scores using Numerical Rating Scale (NRS). Data are presented as mean (±) SD in response to treatment from baseline to weekly and monthly follow-ups. NRS = Numerical Rating Score. D = Day. EGF = Epidermal Growth Factor

Pain scores (NRS) used to monitor M TMD subjectively at baseline,7, 14, 21 days, 1 month and 3 months for both treatment groups are shown in Fig. 2. Intragroup analyses showed that there was a significant reduction in pain scores in response to both treatment modalities (*P* **< 0.000)** (Table 2). Post-hoc Wilcoxon test (Table 3) showed that there was a significant decrease in pain scores starting from day 7(1st week) in group I (*P* = **0.001**) and in group II (*P* **= 0.002)**. Pain levels reached the lowest peak in response to treatment at 1-month follow-up (*P* = **0.001**) for group I and group II. A relapse in pain was evident at 3 months follow-up; however, for group I this relapse was non-significant (*P* = 0.06) compared to 1-month follow-up. For group II patients this rebound in pain scores was significant compared to 1 month follow-up (*P* = **0.011**) but still did not reach pain scores that were recorded at baseline. For intergroup analyses, non-parametric Mann-Whitney analysis showed that there was no significant difference between the two test groups (PLLT Vs EGF injection).

### Effects of treatment modalities on pain free opening (PFO)

**Fig. 3:**
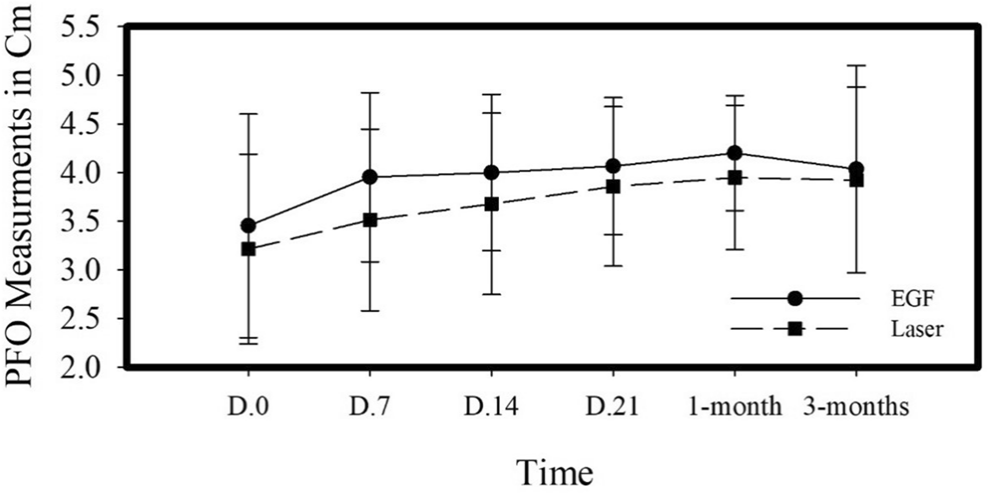
Effect of treatment modalities on Pain Free Opening (PFO). Data are presented as mean and (±) SD of PFO in centimeters in response to treatment at baseline and weekly and monthly follow-ups. PFO = Pain Free Opening. EGF = Epidermal Growth Factor. D = Day

Results of PFO at baseline, 7, 14, 21 days, 1 month and 3 months in response to both treatment modalities are shown in Fig. 3. Intragroup analyses showed that PFO significantly increased in response to treatment in both groups (***P*** < **0.0001)** as shown in Table 2. Post hoc Wilcoxon test (Table 3) showed that PFO started to significantly increase in the 1st follow-up (D7) for group I treatment groups (***P* = 0.016**) and 2nd follow-up (D14) for group II patients (***P* = 0.046**). Pain Free Opening increased significantly reaching maximum peak at 1-month follow-up for group I and group II respectively, (*P* = **0.006**, *P* = **0.011**) compared to baseline. At 3-month follow-up, PFO started to decrease; this decrease was non-significant for group I compared to 1-month follow-up and was still significantly more than PFO recordings at baseline (***P* = 0.008**). A relapse in PFO recordings for group II patients at 3-month follow-up was recorded and recordings were comparable to those at baseline. Intergroup analysis using non-parametric Mann-Whitney test showed that there was no significant difference between the 2 test groups regarding increase in pain free opening.

### Effects of treatment modalities on maximum opening (MO)

**Fig. 4:**
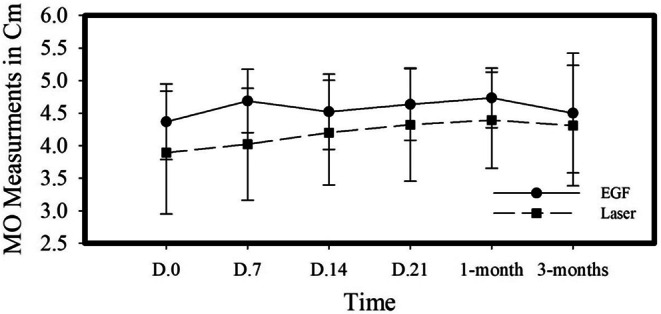
Effect of treatment modalities on Unassisted Maximum Mouth Opening (MO). Data are presented as mean and (±) SD of MO in centimeters in response to treatment modalities at baseline and during weekly and monthly follow-ups. MO = Maximum Mouth Opening. EGF = Epidermal Growth Factor. D = Day

Results of MO at baseline, 7, 14, 21 days, 1 and 3 months are shown in Fig. 4. Intragroup analyses show that there was a significant increase in MO in group I patients receiving PLLT sessions (***P*** **= 0.007**), however in group II patients, MO increase was non-significant. For group I patients, post hoc Wilcoxon -analysis showed that a significant increase in MO was recorded after the 2nd session (D14). At 3 months follow-up, group I patients showed a slight relapse in MO recordings. However, this decrease was non-significant compared to the 1-month follow-up and MO recordings were significantly higher than baseline readings (*P* = **0.03**). Intergroup analysis shows that there is no significant difference in increase of MO between the two test groups.

### Effects of treatment modalities on quality’ of life measured by oral Health Impact Profile (OHIP-14) questionnaire

**Fig. 5:**
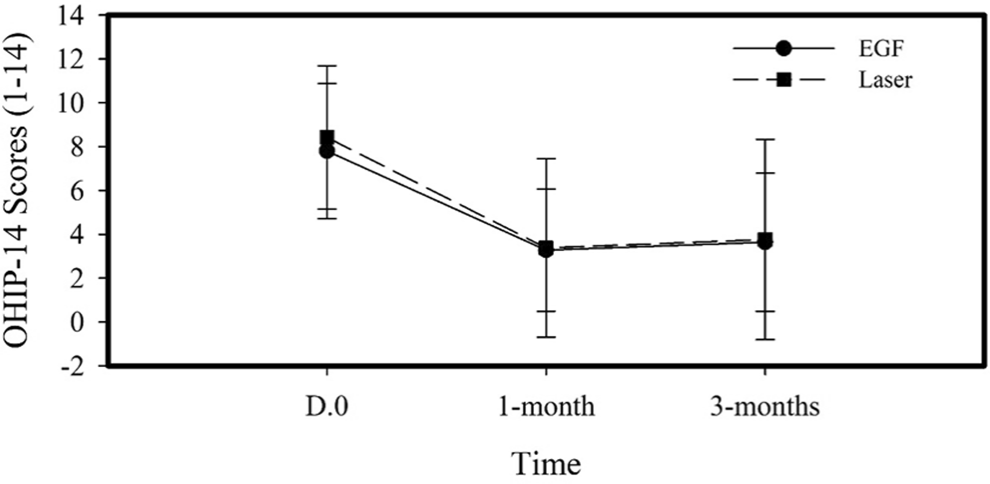
Effect of treatment modalities on quality of life as measured by Oral Health Impact Profile (OHIP-14) questionnaire. Data are presented as mean and (±) SD of OHIP-14 scores in response to treatment modalities at baseline, 1- and 3-month follow-ups. OHIP = Oral Health Impact Profile-14 questionnaire. EGF = Epidermal Growth Factor

Results of OHIP-14 questionnaire scores at baseline, 1 month and 3 months are shown in Fig. 5. Intra-group analysis showed a significant improvement in quality of life in both treatment groups in response to treatment (*P* = **0.0001**) (Table 2). Post hoc Wilcoxon analysis showed that the quality of life significantly improved at 1-month and 3-months follow-ups compared to baseline (Table 3). However, there was no significant difference between 1 month and 3 months in both groups (Table 3). Intergroup analysis shows that there is no significant difference between the two treatment modalities regarding impact on quality of life.

**Table 2:** Results from the nonparametric Friedman’s analyses used to test for intragroup significant variations in the repeated measured mean of different parameters over time

Parameters	Friedman’s Test					
	Group I PLLT (*N* = 13)			Group II EGF (*N* = 14)		
	Chi-square	df	*P*	Chi-square	df	*P*
NRS	34.08	5	**< 0.0001**	44.95	5	**< 0.0001**
PFO	22.27	5	**< 0.0001**	14.86	5	**0.011**
MO	16.09	5	**0.007**	8.99	5	0.109
OHIP-14	19.60	2	**< 0.0001**	19.88	2	**< 0.0001**

PLLT: Piano Level Laser Therapy, EGF = Epidermal Growth Factor, NRS = Numerical Rating Score, PFO = Pain Free Opening, MO = Maximum opening, OHIP-14 = Oral Health Impact Profile, N = the number of patients, df = degree of freedom, and ***P*** = probability values. The bold values indicate significance at α level ≤ 0.05

**Table 3:** Results of post-hoc Wilcoxson test for significant posterior differences in mean data of the measured parameters over time

**Parameter**	**Group I PLLT**											
	**D0 vs. D7**		**D0 vs. D14**		**D0 vs. D21**		**D0 vs. 1-m**		**D0 vs. 3-m**		**1-m vs. 3-m**	
	**Z**	***P***	**Z**	***P***	**Z**	***P***	**Z**	***P***	**Z**	***P***	**Z**	***P***
NRS	-3.19	**0.001**	-3.20	**0.001**	-3.22	**0.001**	-3.20	**0.001**	-3.07	**0.001**	-1.89	0.06
PFO	-2.4	**0.016**	-1.98	**0.048**	-2.54	**0.011**	-2.73	**0.006**	-2.65	**0.008**	-0.21	0.83
MO	-1.89	0.59	-2.04	**0.041**	-2.41	**0.016**	-2.22	**0.026**	-2.16	**0.03**	-0.82	0.41
OHIP	***	***	***	***	***	***	-3.19	**0.001**	-2.94	**0.003**	-0.80	0.32
**Group II EGF**												
**Parameter**	**D0 vs. D7**		**D0 vs. D14**		**D0 vs. D21**		**D0 vs. 1-m**		**D0 vs. 3-m**		**1-m vs. 3-m**	
	**Z**	***P***	**Z**	***P***	**Z**	***P***	**Z**	***P***	**Z**	***P***	**Z**	***P***
NRS	-3.09	**0.002**	-3.19	**0.001**	-3.31	**0.001**	-3.31	**0.001**	-3.18	**0.001**	-2.55	**0.011**
PFO	-1.95	0.051	-1.99	**0.046**	-2.21	**0.027**	-2.54	**0.011**	-1.69	0.092	-1.13	0.257
OHIP	***	***	***	***	***	***	-3.31	**0.001**	-3.08	**0.002**	-0.45	0.653

PLLT: Piano Level Laser Therapy, EGF: Epidermal growth factor, D: day, m: months, NRS: Numerical Rating Score, OHIP-14: Oral Health Impact Profile-14, Z = z- score of the test statistic, and *P* = probability, the bold values indicate significant differences at α level ≤ 0.05. The * indicates an unaffordable test

### Correlation between pain scores, objective clinical outcomes, quality of life, and age

Results of Spearman correlation, shown in Table 4, revealed a strong negative relation between pain scores (NRS) and both pain free opening and maximum opening (*P* **< 0.0001**). The relationship between NRS and OHIP-14 data was significantly highly positive (*r* = 0.699 and *P* < 0.0001). It was also found that as pain score decreases, the quality of life of patients suffering from M TMD improves (*P* **< 0.0001**), as evidenced by a decreased OHIP-14 score. A positive relationship between pain free opening and maximum mouth opening (*P* **< 0.0001**) was also detected. A relation between quality of life and objective clinical outcomes was documented; as PFO and MO increase, quality of life as measured by OHIP-14 score improves (*P* **< 0.0001**). Another finding is the relation between the age of the patients and both PFO and MO. It was found that pain free opening (*P* = **0.0040**) and maximum mouth opening (*P* **< 0.0001**) both decrease with age.

**Table 4:** Correlation between different measured parameters: Pain scores, Pain Free opening, Maximum Mouth opening, quality of life and age of participants

Parameters	*r*	*P*
NRS vs. PFO	-0.569	**< 0.0001**
NRS vs. MO	-0.452	**< 0.0001**
NRS vs. OHIP	0.699	**< 0.0001**
NRS vs. Age	0.191	0.088
PFO vs. MO	0.807	**< 0.0001**
PFO vs. OHIP	-0.507	**< 0.0001**
PFO vs. Age	-0.314	**0.0040**
MO vs. OHIP	-0.454	**< 0.0001**
MO vs. Age	-0.451	**< 0.0001**
OHIP vs. Age	0.133	0.236

Spearman Rho correlation results between the measured parameters over the monthly follow-up at significant ά level less than or equal 0.05. r = correlation coefficient, *P* = probability, NRS = Numerical Rating score, PFO = Pain Free Opening, MO = Maximum Opening, OHIP = Oral Health Impact Profile Questionaire-14, and Age. The bold values indicate significant correlations between the measured parameters

## Discussion

Myogenic TMD (M TMD) being the most common cause to TMD, is characterized by painful muscles of mastication, with the temporalis and masseter muscles being the most involved [41]. In this clinical trial two treatment modalities were evaluated for the treatment of M TMD in terms of pain relief, pain free opening, maximum mouth opening and impact on quality of life. The selection of laser as a physiotherapeutic modality versus ready-made growth factor chair side injection was based on the urge to replace invasive treatment options with more minimally to non-invasive treatment modalities with minimal pain and downtime. The use of lasers and allogenic growth factor injection for M TMD pain was found appealing to both patient and clinician. The results of this clinical trial have shown that both treatment modalities have a positive impact on pain reduction and quality of life, as measured by NRS and OHIP-14, respectively. PLLT significantly increased both PFO and MO, whereas EGF injection only increased PFO significantly.

M TMD is more prevalent among females during child-bearing years with a female-to-male ratio 2:1 [41]. More than 85% of the participants in this clinical trial were females with a mean age which coincides with child-bearing years. Palmer and Dunher [42] stated that females are at a higher risk of developing chronic TMD. This higher prevalence and persistence of TMD pain in female patients could be explained by difference in peripheral and central pain processing between males and females as well as sex hormones, where estrogen increases pain perception and sensitivity among females [43].

Laser photo-biomodulation has been widely used to treat painful orofacial conditions and diseases. High intensity laser therapy (HILT) has been recently introduced for treatment of many painful joints and muscles [44, 45]. The use of HILT in combination with exercises was tested for treatment of knee osteoarthritis and was found to be more effective than low level laser therapy (LLLT) in combination with exercises and more than exercises alone [44]. HILT with a pulsed Nd-YAG laser was tested on patients suffering from myogenic TMD pain and compared to placebo HILT [46]. The study showed that HILT was an effective pain treatment modality in myogenic TMD patients and improved their quality of life [46]. In this clinical trial the use of pulsed 1064 nm Nd-YAG laser source was preferred over LLLT because it can penetrate deeper tissue and stimulate wider areas thus working on different levels namely mitochondrial oxidative response, vascular dilatation and collagen stimulation in muscles and tendons [44, 45]. This is the first clinical trial to use Nd-YAG laser in PIANO^®^ mode (two levels of HILT) for myogenic TMD pain allowing the delivery of long pulsed and low fluence laser energy deeper and to a wider area with minimal pain and burning sensation. It also allows non-thermal biomodulation and thermal pain management. The use of Genova handpiece that was specially developed for Fotona’s LightWalker laser system allowed inducing highly effective pain reduction. The handpiece has a large spot size that is stable regardless of the distance from the patient with a unique collimated homogeneous beam profile of Nd: YAG laser light [47].

The results of this clinical trial showed that patients treated with PLLT showed a significant reduction in pain scores starting day 7 (after the first session), pain scores kept on decreasing significantly till the 1st month. Despite the slight rebound in pain scores at 3 months follow-up, pain scores at 3 months were still significantly lower than baseline which gives an indication about the success of the use of PLLT as a non-invasive long term treatment modality for myogenic TMD pain. These results are consistent with those of Ekici et al. that showed a significant reduction in pain scores at 4 and 12 weeks follow-up compared to baseline in myogenic TMD patients treated by Nd-YAG laser [46]. Compared to LLLT; PLLT can offer the advantage of a more rapid relief of pain and faster response. Al-Quisi et al. [48], when comparing LLLT to light therapy showed that patients receiving LLLT had a significant reduction in pain scores compared to placebo after the 3rd visit (3rd week) unlike the results of this current trial that showed a significant reduction in pain after the 1st visit.

Many needling therapies have been tried for myogenic TMD pain. EGF has been well documented for its positive role in wound healing, burns, diabetic foot ulcerations, many dermatological conditions and aesthetic applications [49]. However, EGF role in muscle fiber regeneration and myogenic pain solely has not been extensively studied despite being one of the platelet-associated growth factors found in PRP [49–51]. To the best of our knowledge, this is the 1st clinical trial to use an exogenous ready-made EGF low molecular weight polypeptide for treating myogenic TMD pain. The use of an allogenic, ready-made source of growth factors like DERMAQUAL EGF spares the limitations associating the use of autologous PRP or PRF related to preparation protocol or donor blood. Preparation protocol limitations include lack of standardization in centrifugation force, time, number and added anticoagulants or other additives [52]. Donor issues include discrepancies in patient age, gender, disease status, and platelet count [53]. The use of allogenic EGF is less invasive compared to PRP because there is no need for withdrawal of large blood samples from patients.

The results of this clinical trial revealed that pain was significantly reduced at day 7, i.e., after the 1st injection and continued to decrease significantly every week till the 1-month follow-up (lowest peak). These findings are consistent with Agarwal et al., who found a significant reduction in pain scores at 2 and 4 weeks after PRP injection [54]. However, patients injected with EGF used in this study showed a slight relapse in pain scores at 3-months follow-up compared to 1-month but were still significantly lower than baseline. Agarwal et al., showed a rebound in pain scores at 3 months compared to 4-weeks follow-up, however; at 6-months follow-up pain scores decreased again compared to 4-weeks [54]. This suggests the possibility of a dual impact of growth factor injection on M TMD pain; a short-term effect by directly acting on pain and a long-term effect that targets regeneration of muscle fibers, healing and secondarily pain. This highlights the urge for longer follow-up periods at 6-months and 1-year to assess the effectiveness of EGF injection. Kao et al., have shown that the use of anti-adhesive nanofibers loaded with lidocaine and EGF offered sustained postoperative pain relief and accelerated wound healing. The role of EGF in wound healing was explained by the role of EGF on keratinocytes, endothelial cells and fibroblasts thus allowing regeneration which could also be responsible for recovery in patients suffering from M TMD [55]. Furthermore, EGF has been found to competitively block N-Methyl-D-Aspartate (NMDA) receptors that play a role in pain sensitization in chronic M TMD chronic M TMD [56, 57].

To assess the impact of the two tested treatment modalities on mandibular range of motion, both pain free opening and maximum unassisted opening (even if associated with pain) were measured. These two vertical plane procedures have proved excellent reliability and thus are excellent tools to monitor joint function over time [58]. Results of this study showed that both treatments had a positive impact on pain free opening. However, PLLT showed a rapid response compared to EGF injection, where patients enrolled to PLLT group showed a significant increase in PFO at day 7 (first session), whereas patients enrolled to EGF group showed a significant increase in PFO at day 14. This could be explained by the fact that HILT primary mode of action is through affecting cutaneous nerve endings, causing reactive vasodilation and thus an immediate analgesic response [44]. Another mode of action is through the stimulatory effect of HILT on cellular and tissue levels; stimulating mitochondrial ATP production, secretion of beta-endorphins and thus creating an anti-inflammatory and analgesic medium [44]. The relatively delayed response in PFO recordings in group II might be due the intramuscular post-injection pain where this is a minimally invasive procedure compared to group I patients who were treated in a totally non-invasive manner. At 1-month follow-up, patients enrolled in both groups showed the maximum PFO readings compared to baseline as shown in Fig. 3. PFO at 1 month follow up was significantly higher than that recorded at baseline in both test groups.

Based on the results of this clinical trial, PLLT sessions showed longevity in pain relief compared to EGF injection. This is manifested by sustained pain scores and PFO readings at 3-months compared to 1-month follow-up and baseline in PLLT group. Whereas group II patients (EGF) showed a significantly higher pain score and declined PFO reading compared to 1-month follow-up and that was comparable to baseline readings. Additionally, MO showed a significant increase only in group I patients at the 2nd PLLT session compared to group II. This gives PLLT sessions another privilege over EGF injections, but could be still attributed to the higher MO recordings in group II patients at baseline compared to group I.

Quality of life is an important parameter to consider when evaluating the effectiveness of any treatment modality, especially in chronic painful conditions like myogenic TMD. The selection of OHIP-14 for quality-of-life assessment came from the fact that it is well recognized internationally, concerned with oral functions and gives an idea about multiple domains, functional limitation, physical pain, psychological discomfort, physical incapacity, psychological incapacity, social incapacity and handicap [59]. The results of this clinical trial showed a significant improvement in quality of life at 1- and 3-month follow-up in response to both treatment modalities indicating the effectiveness of both treatments on short and long terms. This clinical trial has also shown that there was no significant difference between the two test groups at baseline, 1 month and 3 months follow up. Patients enrolled in both groups experienced no side effects or complications.

It was found that pain scores were negatively correlating with both PFO and MO and positively correlating with OHIP-14 scores (where lower scores indicated better quality of life). This reflects the impact of pain on both TMJ function and patient quality of life. The negative correlation between pain and both MO and PFO can be explained by the fact that patients with myogenic TMD do not have an actual limitation in mouth opening due to any pathologic factor related to the joint but rather, restriction in mouth opening is only a prophylactic response from the patient to avoid pain, as myogenic pain increases upon muscle stretching [60]. This can also be supported by the finding that most of the patients had MO within normal limits.

Despite the proved effectiveness of the two treatments on M TMD chronic pain, yet the sample size was small, and patient were followed-up up to 3 months only. Future randomized clinical trials on larger scale and longer follow-up periods are recommended to investigate the long-term impact of treatments on M TMD pain. Also, several treatment protocols need to be trialed to consider the optimum number of treatment and maintenance sessions as well as the different doses per injection point for EGF injection. Further studies monitoring different pain and inflammatory markers in response to treatment can help understand different underlying mechanisms of action on relieving M TMD pain.

In conclusion, based on the results of this clinical trial both modalities can offer effective non- to minimally invasive treatment options for patients with refractory chronic M TMD pain. The patients enrolled in this clinical trial reported no side effects with either treatment. The clinical visit was short ranging from 10 to 15 min with no-to-minimal pain encountered by patients. There was also no downtime associated with PLLT sessions, and patients reported immediate relaxation of muscles. For EGF injection, mild postoperative pain was reported in some of the patients, which disappeared spontaneously within the first 48 h post injection. PLLT offered slightly better longevity compared to EGF injection. However, EGF injection was less expensive than PLLT sessions.

## Electronic supplementary material

Below is the link to the electronic supplementary material.

Supplementary Material 1

## Data Availability

No datasets were generated or analysed during the current study.
