# Supplementary material for: Piano level laser therapy versus epidermal growth factor injection for painful myogenic temporomandibular disorder (a randomized clinical trial)
Source: Clin Oral Investig. 2025 Feb 6;29(2):118. doi: 10.1007/s00784-025-06189-5 (PMC11802707; doi:10.1007/s00784-025-06189-5)
Supplement: Supplementary file 1 — Supplementary Material 1 [file 784_2025_6189_MOESM1_ESM.docx]

| Statistic measure | Weekly Follow-up | | | | | | | | | | | |
| --- | --- | --- | --- | --- | --- | --- | --- | --- | --- | --- | --- | --- |
|  | Group I PLLT (N= 13) | | | | | | Group II EGF (N= 14) | | | | | |
|  | Age (28.23±7.07) | | | | | | Age (27.30±7.25) | | | | | |
|  | D0 | D7 | | D14 | | D21 | D0 | D7 | | D14 | | D21 |
| NRS | 7.85±1.52 | 4.27±2.76 | | 3.00±2.80 | | 1.92±2.25 | 7.36±1.82 | 4.68±3.23 | | 3.79±2.67 | | 3.36±2.82 |
| PFO | 3.23±1.01 | 3.55±0.96 | | 3.69±0.97 | | 3.85±0.85 | 3.52±1.16 | 3.95±0.90 | | 4.00±0.83 | | 4.07±0.73 |
| MO | 3.92±0.98 | 4.06±0.88 | | 4.22±0.83 | | 4.35±0.90 | 4.40±0.59 | 4.66±0.50 | | 4.49±0.58 | | 4.61±0.56 |
|  | Monthly Follow-up | | | | | | | | | | | |
|  | Group I PLLT (N= 13) | | | | | | Group II EGF (N= 14) | | | | | |
|  | Age (28.23±7.07) | | | | | | Age (27.30±7.25) | | | | | |
|  | D0 | | 1-m | | 3-m | | D0 | | 1-m | | 3-m | |
| NRS | 7.85±1.52 | | 1.00±1.92 | | 3.23±3.09 | | 7.36±1.82 | | 1.71±1.73 | | 3.57±3.15 | |
| PFO | 3.23±1.01 | | 3.946±0.77 | | 3.92±0.95 | | 3.52±1.16 | | 4.21±0.61 | | 4.04±1.06 | |
| MO | 3.92±0.98 | | 4.39±0.77 | | 4.31±0.93 | | 4.40±0.59 | | 4.71±0.469 | | 4.50±0.92 | |
| OHIP | 8.08±3.12 | | 3.38±4.07 | | 3.77±4.57 | | 7.79±3.19 | | 3.64±3.15 | | 3.64±3.15 | |

Appendix 1: Mean and standard deviations (±) of the measured parameters for 27 study participants between the two treatment modalities over weekly and monthly follow-up.

Abbreviations: PLLT: Piano low level laser therapy, EGF = Epidermal growth factor, D= day, m= month(s), NRS= Numerical Rating Score, PFO= Pain Free Opening, MO= Maximum Opening, OHIP= Oral Health impact Profile-14.
